# Lasting Effects of Low-Frequency Repetitive Transcranial Magnetic Stimulation in Writer’s Cramp: A Case Report

**DOI:** 10.3389/fnhum.2019.00314

**Published:** 2019-09-10

**Authors:** Antonino Naro, Luana Billeri, Simona Portaro, Placido Bramanti, Rocco Salvatore Calabrò

**Affiliations:** Behavioral and Robotic Neurorehabilitation Unit, IRCCS Centro Neurolesi Bonino Pulejo, Messina, Italy

**Keywords:** writer’s cramp, repetitive transcranial magnetic stimulation, left premotor cortex, non-invasive neuromodulation, sensorimotor plasticity, handwriting

## Abstract

The treatment of writer’s cramp (W’sC) is essentially based on the use of botulinum toxin. However, additional treatments are sometime required to prolong the effects of the toxin, compensate for its progressive loss of efficacy in some subjects, and re-educate handwriting (e.g., rehabilitation strategies). Low-frequency repetitive transcranial magnetic stimulation (rTMS) has been employed to improve W’sC, but with short-lasting and controversial outcomes. We report on the effects of a long-lasting low-frequency rTMS paradigm on W’sC symptoms. A 25-year-old male with a diagnosis of simple W’sC was enrolled in the study. He underwent an objective assessment using the Writer’s Cramp Rating Scale (WCRS) and the 1-min writing test. Further, we recorded muscle activation of the upper limb during handwriting using an EMG wireless system. The patient was provided with 1,200 biphasic magnetic pulses delivered at 1 Hz over the left premotor cortex (PMC), 15 times scheduled every 2 days, thus covering a period of 5 weeks, followed by 10 days of rest. This block of stimulations was practiced other four times, for a period of 6 months. The patient showed a gradual clinical improvement with the progression of the treatments. W’sC symptoms totally disappeared and all the clinical scores showed a significant improvement after rTMS completion. Such improvement lasted up to 1 year after the end of the treatment. Moreover, we detected a long-lasting improvement in sensorimotor plasticity as measured by a paired associative stimulation protocol. Our case suggests that the long-lasting application of 1 Hz rTMS to PMC is a safe and potentially valuable tool to improve W’sC symptoms enduringly, probably by reverting maladaptive plasticity mechanisms within the sensory-motor areas of the hemisphere contralateral to the dystonic hand.

## Introduction

Writer’s cramp (W’Sc) is a task-specific dystonic movement disorder characterized by involuntary cramping of muscles of the hand, forearm, or upper arm selectively triggered by writing (Sheehy and Marsden, 1982; Stahl and Frucht, 2016), with a noticeable impairment of writing. Typically, the subject holds the pen with exaggerated effort during handwriting, while the upper limb takes particular postures, such as wrist flexion or extension, arm elevation, and shoulder abduction. Writing shows a progressive loss in speed and accuracy of the characters, which grow larger, smaller, and longer, with a loss of word alignment. The abnormal upper limb posture is to compensate that of hand (Quartarone and Hallett, 2013). The treatment of W’sC is essentially based on the use of botulinum toxin. Additional treatments are however required to prolong the effects of the toxin, compensate for its progressive loss of efficacy in some subjects, and re-educate handwriting (i.e., rehabilitation strategies). The rationale for choosing an add-on strategy in dystonia should be guided by the pathophysiology of the disease (Quartarone et al., 2017). Indeed, dystonia is characterized by: (i) a loss of inhibition at different levels of the central nervous system; (ii) a maladaptive (excessive) plasticity within sensorimotor areas; and (iii) an altered sensorimotor integration and sensorimotor plasticity (Ridding et al., 1995; Quartarone et al., 2003; Bäumer et al., 2007; Hallett, 2011; Quartarone and Hallett, 2013; Conte et al., 2019). Therefore, strategies aimed at restoring the maladaptive plasticity mechanisms within sensorimotor areas in patients with W’sC may be useful. About that, recent studies examining the effects of repetitive transcranial magnetic stimulation (rTMS) and transcranial direct current stimulation to augment current rehabilitation techniques offered some encouraging results (Cho and Hallett, 2016; Erro et al., 2017; Quartarone et al., 2017). Specifically, rTMS consists in the delivery of magnetic pulses at a certain frequency and intensity on a brain area. The modulation effect can range from inhibition to facilitation depending on the stimulation parameters used. Further, rTMS can induce effects on cortical excitability that outlast the stimulation itself. By means of these effects, rTMS can modulate the sensorimotor plasticity, thus potentially fostering the rehabilitative outcomes in W’sC (Cho and Hallett, 2016; Erro et al., 2017; Quartarone et al., 2017). Nonetheless, there are a few reports on the efficacy of rTMS on W’sC, and these studies are limited to about 1–2 weeks of treatment and lack of an adequate follow-up (Erro et al., 2017; Quartarone et al., 2017). The aim of this study was to preliminary assess the safety and usefulness of a 1 Hz rTMS paradigm delivered over the left premotor cortex (PMC) three times a week for 3 months to reduce W’sC symptoms. The study illustrates how efficiently the rTMS paradigm provided the patient with a nearly complete resolution of W’sC symptoms for 1 year. This work also reviewed W’sC cases with regard to rTMS treatment. Cases of long-lasting 1 Hz rTMS paradigm applied to patents with W’sC have not been previously reported in the literature.

## Patient and Methods

All procedures performed in our study involving human participants were in accordance with the ethical standards of the institutional and/or national research committee and with the 1964 Helsinki Declaration and its later amendments or comparable ethical standards. The Local Ethics Committee approved the present study (IRCCSME 39/18). The patient provided his written informed consent to study participation and publication.

**Figure 1:**
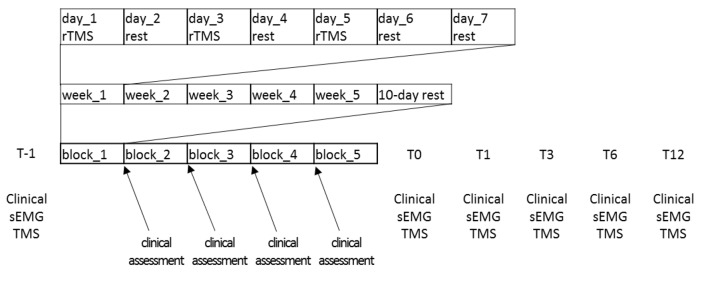
Experimental procedure outline.

A 25-year-old, right-handed male with a diagnosis of simple W’sC (i.e., other acts of dexterity were not impaired) was enrolled in the study. His family history was negative for essential tremor or dystonia. The patient was a university student and he did not suffer from any other neurological and orthopedic deficits. He has had symptoms exclusively in the right hand and arm for about 6 years. Prolonged rest (including holiday and vacation) did not influence his symptoms. He previously refused treatments with antispastic, myorelaxant, or botulinum toxin, and only practiced retraining/physiotherapy training programs aimed at improving W’sC (the last one occurred more than 6 months before our observation). As he began to not tolerate the symptoms anymore, he came to our observation to be treated with rTMS. Before enrollment (T-1), he underwent an objective assessment using the Writer’s Cramp Rating Scale (WCRS; that rates dystonic posturing of the elbow, wrist, and fingers, latency of dystonia and tremor, and writing speed; range = 0–30, higher is worse) and the 1-min writing test (1MWT, in which the patient has to write the sentence “*le stelle brillano*”—the stars are shining- at least 12 times within 1 min), and a subjective assessment using self-evaluation of handwriting impairment (HI) and pain intensity (PI), using visual analog scale. We also confirmed the diagnosis of W’sC according to the current diagnostic criteria (Hallett, 2006; Albanese et al., 2013). The functional (psychogenic) nature of the movement disorder shown by the patient was also excluded (Ganos et al., 2014; Hallett, 2016). The patient was negative at genetic screening (formerly performed), given the very early onset of the W’sC. The patient also underwent a surface EMG recording during handwriting and a TMS assessment. We recorded muscle activation of upper limb muscles during handwriting using an EMG wireless system (FreeEMG1000 system; BTS Bioengineering, Milan, Italy). Surface myoelectric signals were sampled at 1,000 Hz from eight muscles of the right upper limb: deltoid, pectoralis major, biceps, triceps, flexor carpi radialis (FCR), extensor digitorum communis (EDC), abductor pollicis brevis (APB), and first dorsal interosseous (FDI). After careful preparation of the skin, the bipolar adhesive surface electrodes were placed over the muscle belly in the direction of the muscle fibers according to the European recommendations for surface electromyography (SENIAM) to ensure repeatable electrode placement over the treatment (Blanc and Dimanico, 2010; Nishihara and Isho, 2012; Ghapanchizadeh et al., 2016). The EMG signals were analyzed using the Smart Analyzer software (Version 1.10.469.0; BTS, Milan, Italy) for root-mean-square (RMS) calculation (a temporal parameter estimating muscle activation), to investigate muscle activation pattern modified by rTMS intervention (Boe et al., 2008). Using TMS, we assessed the effects of 1 Hz rTMS paradigm delivered over the left PMC on various measures of motor cortical excitability and sensorimotor plasticity. We first measured the resting (RMT) and active motor threshold (AMT) from left primary motor area (M1). RMT was defined as the minimum intensity that evoked a peak-to-peak motor evoked potential (MEP) of 50 μV in at least 5 out of 10 consecutive trials in the relaxed APB muscle. AMT was defined as the minimum intensity that elicited a reproducible MEP of at least 200 μV in the tonically contracting APB muscle in at least 5 out of 10 consecutive trials. The patient maintained a force level of approximately 10%–15% of maximum force during measurements of the AMT (Rossini et al., 1999; Quartarone et al., 2006). We then recorded MEP amplitude from both FDI and APB muscles at rest and during voluntary contraction to estimate the duration of the cortical silent period (CSP). To this end, TMS pulses with a monophasic pulse configuration were given to the left M1 using a standard 90 mm figure-of-eight coil connected with a high-power Magstim200 stimulator. The center of the coil was located over the motor hot spots for the stimulation of the contralateral APB and FDI muscles. The handle of the coil pointed 45° postero-laterally with respect to the midline. Fifteen MEPs were recorded (pulse stimulations applied at 0.1 Hz, gain: 200–500 μv/div, filter: 20 Hz–2 kHz). For each muscle using a stimulus intensity of 120% of RMT. The average peak-to-peak amplitude of the MEP was taken as a measure of corticospinal excitability. Concerning CSP, we measured the peak-to-peak amplitude of 10 consecutive MEPs during slight tonic contraction of the right APB muscle at ~15% of maximum force level. Audiovisual feedback of ongoing EMG activity was provided to ensure a constant force level. Stimulus intensity was identical to the MEP probing. For CSP measurements, EMG traces were rectified but not averaged. The duration of the CSP was measured in each trial and defined as the time from the onset of the MEP to reappearance of sustained EMG activity (Orth and Rothwell, 2004). The CSP duration is a marker for the excitability of long-lasting (presumably GABA_B_ergic) intracortical inhibition (Siebner et al., 1998; Werhahn et al., 1999). Surface EMG data were collected using silver chloride disk electrodes placed on the target muscles in a bipolar belly-tendon montage with a bandpass filter 20 Hz–2 kHz (Cadwell Laboratory, Kennewick, WA, USA). Sensorimotor plasticity from the M1 contralateral to the affected limb was probed by measuring the effects of a rapid paired associative stimulation (rPAS) protocol on APB and FDI MEP amplitude changes (Quartarone et al., 2006). rPAS consisted of 600 pairs of stimuli which were continuously delivered at 5 Hz to the left M1. Each pair of stimuli consisted of an electrical conditioning stimulus given to the right median nerve followed by a biphasic TMS pulse given to the left M1. We used an interstimulus interval of 25 ms (Wolters et al., 2003; Ghapanchizadeh et al., 2016). Electrical conditioning stimuli consisted of square wave pulses provided through a bipolar electrode (Digitimer D-160 stimulator; Digitimer Limited, Welwyn Garden City, Herts, UK). The cathode was located proximally and the pulse-width was 500 μs. rTMS was given through a standard 90 mm figure-of-eight-shaped coil connected to a Magstim Rapid stimulator (Magstim Company Limited, Whitland, Dyfed, UK). The intensity of the electrical stimulus was set at twice the sensory threshold, while the intensity of TMS was individually adjusted to 90% of AMT. The topographic specificity of rPAS aftereffects was assessed by measuring MEP amplitude from both FDI and APB (Ziemann, 2018). The treatment protocol consisted of 1,200 biphasic magnetic pulses delivered in a single session at 1 Hz over a point sited at 2 cm anterior and 1 cm medial to the previously defined hotspot for FDI activation (Fink et al., 1997; Schluter et al., 1998; Murase et al., 2005; Borich et al., 2009). We adopted a 90 mm figure-of-eight coil (centered tangentially on the scalp with its handle pointing in a posterior direction and laterally at an angle of approximately 45° away from the midline) wired to a Magstim Rapid^2^ magnetic stimulator (Magstim Company Limited, Whitland, Dyfed, UK). The stimulation intensity was set at 90% of AMT. The patient was provided with blocks of 15 rTMS sessions, practiced three times a week (scheduled every 2 days), thus covering a period of 5 weeks, followed by 10 days of rest. This block of stimulations was practiced five times, thus covering a period of nearly 8 months (Figure 1). To ensure constant stimulation conditions across sessions, a fixation unit with an integrated head holder was built, upon which a flexible coil holder was mounted. The individual wore earplugs during the rTMS, he was seated in a comfortable armchair with the head fixed to a head holder by a headband. The coil position was drawn on the scalp, and constant coil position was continuously monitored throughout the experiment. Hotspot and stimulation intensity were checked before starting every TMS session.

## Results

The patient was clinically and electrophysiologically re-evaluated immediately after 0 (T0), and after 1 (T1), 3 (T3), 6 (T6), and 12 months (T12) the end of the protocol. Given that this was a case report, a semi-quantitative scale was used for rating clinical and electrophysiological measure changes at each T after rTMS compared to T-1 (negligible <±20%, mild ±20%–40%, moderate ±40%–70%, large ±70%–100%, very large >±100%). At T-1, the patient complained of a WCRS score of 13, a low HF score, and a barely appreciable PI. He reported a great effort during handwriting at the 1MWT, yet immediately after he started to write. He wrote significantly slowly and with a low mean stroke frequency. Further, writing movements were irregular in terms of velocity, indicating an impaired fluency of handwriting. The dystonic pattern during handwriting was characterized by wrist flexion with ulnar deviation, flexion of thumb and index, elbow flexion and shoulder abduction. EMG data during handwriting showed multiple 4–5 Hz bursts of co-contraction of FCR/EDC and APB/FDI muscles. CSP duration was 98 ms, MEP amplitude increase induced by rPAS (baseline value 0.8 mV from both the APB and FDI) was very large (Table 1). Further, it lacked of topographic specificity, i.e., MEP amplitude showed a very large increase in both the APB and FDI muscles. We measured only WCRS and the 1MWT following each block of rTMS. The patient clinically improved yet after the first block of rTMS treatment. However, such improvement totally disappeared after 10 days, when the patient had to begin the next rTMS block. The patient showed a further improvement following the second rTMS block and retained some improvement at the beginning of the third block. W’sC symptoms totally disappeared already after the fourth rTMS block, and all the clinical scores showed a large improvement. EMG pattern of muscle activation during handwriting did not show the co-contraction identified at baseline. CSP duration showed a very large increase, whereas MEP amplitude increase following rPAS had a very large reduction and clearly improved in topographic specificity (i.e., MEP increased only in APB muscle). Clinical improvement and the modulation of rPAS aftereffects lasted up to T12. CPS duration increase lasted up to T1, and + then mildly decreased up to T12, remaining however largely below T-1 values (Table 1).

**Table 1:** This table reports the clinical and electrophysiological outcomes and the times (T) of assessment.

		T-1 (1b)	1st 15-session block	1a	10-day rest	2b	2nd 15-session block	2a	10-day rest	3b	3rd 15-session block	3a	10-day rest	4b	4a	10-day rest	5b	5th 15-session block	T0 (5a)	T1	T3	T6	T12
WCRS		13		6		13		6		6		1	1		1		1		1	1	1	1	1
1MWT		6		10		6		8		6		9		9	11		11		13	12	12	13	13
HI		8																	2	1	1	0	0
PI		1																	0	1	0	0	0
CSP (% T-1)		98 ms + 100%																	127	128	117	112	111
rPAS aftereffects (%unconditioned	APB	249																	162	175	182	165	142
MEP−0.8 mV)	FDI	268																	105	104	97	104	105
RMT (%SO)	APB	45																	45	46	47	46	44
	FDI	45																	47	47	46	46	46
AMT (%SO)	APB	38																	40	40	39	41	39
	FDI	40																	38	38	39	38	38

*Only WCRS and 1MWT were assessed also before (b) and after (a) each of the rTMS blocks (i.e., 1b, 1a, 2b, 2a, and so on), which were separated each other by a 10-day period of rest. Legend: Writer’s Cramp Rating Scale (WCRS), 1-min writing test (1MWT), handwriting impairment (HI), pain intensity (PI), resting motor threshold (RMT), motor evoked potential (MEP), active motor threshold (AMT), cortical silent period (CSP), rapid paired associative stimulation (rPAS), abductor pollicis brevis (APB), first dorsal interosseous (FDI), stimulator output (SO), nv normative values*.

## Discussion

We report on the case of a young man with W’sC who was provided with a long-lasting 1 Hz rTMS paradigm over the left PMC, practiced three times a week for 3 months, with a nearly complete resolution of W’sC symptoms. Specifically, our patient complained of a significant W’sC symptoms relief yet after the third rTMS block, which further improved after treatment completion and lasted up to 1 year. As far as we know, this is the first report on long-lasting therapeutic effects of rTMS treatment on W’sC symptoms.

The effectiveness of rTMS into treating W’sC has been comprehensively evaluated by some studies (Erro et al., 2017; Quartarone et al., 2017). Indeed, there are some reports supporting the efficacy of low-frequency rTMS (i.e., below 1 Hz) in improving W’sC symptoms, including reduced writing pressure, better global clinical score and handwriting performance (Siebner et al., 1999; Murase et al., 2005; Borich et al., 2009; Havrankova et al., 2010). The design of such studies was single/double-blinded and sham-controlled. Daily rTMS sessions, for 1–5 consecutive days, were delivered at 80%–90% of RMT and targeted one among M1 (Siebner et al., 1999; Murase et al., 2005), PMC (Siebner et al., 2003; Murase et al., 2005; Borich et al., 2009; Havrankova et al., 2010; Kimberley et al., 2013, 2015), supplementary motor area (Murase et al., 2005), and primary sensory area (S1; Havrankova et al., 2010). All clinical effects were paralleled by a normalization of altered cortico-cortical inhibition and prolongation of the CSP (Siebner et al., 1999), prolongation of the CSP only after PMC stimulation (Murase et al., 2005; Borich et al., 2009), and increased task-related BOLD signal in superior parietal lobule in fMRI (Havrankova et al., 2010). Altogether, these data suggest that inhibition of the PMC (Murase et al., 2005; Borich et al., 2009), M1 (Siebner et al., 1999), and S1 (Havrankova et al., 2010) can provide a therapeutic target for W’sC. Nonetheless, the clinical effects lasted no more than 10–14 days after the treatment completion (Gersner et al., 2011). Negative reports on the clinical effectiveness of low-frequency rTMS are also available (Bharath et al., 2015, 2017; Zhang et al., 2017). The design of such studies were single/double-blinded and sham-controlled. One to five daily rTMS sessions were delivered at 80%–90% of RMT and targeted the dorsal PMC. Notably, the paradigms by Kimberley et al. (2013, 2015) was delivered while the patients were engaged in a motor task not triggering dystonic symptoms or sensorimotor training or non-specific stretching/massage. These studies reported no effects in terms of global clinical score and handwriting performance (Zhang et al., 2017), no additional benefit from sensorimotor retraining, and no significant differences between real and sham rTMS (Bharath et al., 2015, 2017). Nonetheless, there were some neurophysiological changes to acknowledge, including a cerebral blood flow reduction in lateral and medial premotor areas, putamen and the thalamus (Zhang et al., 2017), an increased intracortical inhibition (CSP increase), and a reduced pen grip force (Bharath et al., 2015, 2017). All the above-mentioned studies share the low frequency of stimulation, in keeping with the rationale of inhibiting the cortical motor areas contralateral to the affected side (Siebner et al., 1999, 2003; Murase et al., 2005). On the other hand, they are heterogeneous concerning the study designs, targeted brain areas, and number of rTMS sessions applied. These aspects may account for all these contradicting outcomes. Further, it is likely that the intended inhibitory effect of low-frequency rTMS did not outweigh the abnormally high cortical excitability in dystonia by employing single or few rTMS sessions. Nonetheless, some cumulative but short-lasting effects were obtained (Havrankova et al., 2010).

Thus, one may question that the lasting clinical improvement, we got may have simply depended on a banal cumulative effect of the TMS sessions rather than on a specific action of rTMS on cortical plasticity. However, the clinical effects were already appreciable after the first rTMS block, but these disappeared during the resting period of 10 days. Also, we did not find any clinical improvement by targeting the PMC with the first five sessions of 1 Hz rTMS. Last, the effects lasted for at least 1 year after the end of the treatment. These issues indeed suggest a specific action on cortical excitability to guarantee a so long clinical improvement (Borich et al., 2009; Gersner et al., 2011; Zhang et al., 2017). However, we can only speculate on this issue, as we carried limited electrophysiological measures. PAS is a suitable approach to test sensorimotor plasticity abnormalities related to long-term potentiation (LTP; Stefan et al., 2000; Quartarone et al., 2003, 2006; Cho and Hallett, 2016; Erro et al., 2017), which is a form of neuroplasticity based on neuronal co-firing and, thus, wiring over time (Hebb, 1949). When the ratio of the average post- and pre-PAS MEPs is greater than 1, PAS is said to have induced motor facilitation and strengthened sensorimotor plasticity (Stefan et al., 2000). Our patient reported an abnormally high increase of sensorimotor plasticity following PAS application, as it occurs in patients with organic dystonia (Quartarone et al., 2009). Toning down such an abnormal sensorimotor plasticity can have clinical relevance, i.e., can improve motor symptomatology (Hallett, 2007). About that, it is likely that low-frequency rTMS on PMC can have inhibitory effects on motor excitability and can weak sensorimotor plasticity by affecting cortico-cortical inhibitory networks within M1. This may occur through the short-latency inhibition of the pyramidal-tract neurons that may involve excitatory inputs to superficial inhibitory interneurons in the motor cortex (Tokuno and Nambu, 2000). In other words, rTMS on PMC may raise secondary effects on the GABAergic intracortical elements that sustain ICI/ICF. These postulated effects of PMC onto primary motor cortex are based on the pathways between the premotor and the primary motor cortex electrophysiologically demonstrated in monkeys (Ghosh and Porter, 1988; Tokuno and Nambu, 2000). We can only make a hypothesis on this issue, as we did not provide any specific evidence on the role of GABAergic neurons (like cortico-cortical inhibition or neuroimaging findings), but a consistent potentiation of CSP. Even though ICI/ICF and CSP rely on different subsets of inhibitory neurons (Werhahn et al., 1999), an interaction between these two effects is plausible (Chen et al., 1997a). The mechanisms by which rTMS over PMC has inhibitory effects onto sensorimotor cortex are likely GABA_B_ergic, as suggested by the initial potentiation of CSP, whereas the effects onto intracortical inhibition and facilitation mechanism are reported as less clear (Odergren et al., 1998; Münchau et al., 2002; Bäumer et al., 2003; Rizzo et al., 2003; Lerner et al., 2004; Murase et al., 2005; Hallett, 2007; Borich et al., 2009; Huang et al., 2012; Kimberley et al., 2015; Nordmann et al., 2015). Further, it is likely that rTMS exerts its effects onto GABA_B_ergic neurons within the M1 of both the hemispheres (Reis et al., 2008), thus accounting, at least partially, for the widespread cortical inhibition reported in the literature following PMC low-frequency rTMS. We may speculate that the different temporal extent of modulation of CSP and rPAS aftereffects may depend on homeostatic plasticity mechanism (Thapa and Schabrun, 2018). Therefore, it is conceivable that premotor rTMS influences the excitability of inhibitory projections from the PMC that are normally activated in the CSP. Further, we have to take into account that PAS can also directly affect GABAergic neurotransmission (Sale et al., 2007). The reverting effect of rTMS on CSP duration may suggest the association of GABAergic neurons with rTMS aftereffects in dystonia. Nonetheless, further data are required to confirm that dystonia improved by 1 Hz rTMS for long period owing to neuroplasticity changes that led to the reduction of the excitation overflow, including resting or active MEP amplitudes in hand muscles related with handwriting. Indeed, we recorded MEP amplitude from only two muscles of the hand (APB and FDI) as we yearned for demonstrating whether PMC rTMS may recover the loss of topographic specificity or rPAS aftereffects, which correlates with muscle activation spreading and characterizes patients with organic dystonia (Chen et al., 1997b; Rona et al., 1998; Abbruzzese et al., 2001; Bäumer et al., 2007). About that, it has been reported that PMC rTMS can revert the abnormal reciprocal inhibition in patients with hand dystonia, likely acting on topographic specificity mechanisms within sensorimotor areas (Huang et al., 2004). However, we monitored muscle activation pattern during handwriting before and after rTMS protocol application using surface EMG from some muscles of upper limb involved during handwriting tasks. We found a clear reshape of muscle activations, with a complete resolution of the abnormal co-contraction pattern identified at baseline. Further, writing movements were significantly improved. These findings can partially suggest that dystonia improved by 1 Hz rTMS for long period owing to a reduction of the overflow of excitation within sensorimotor networks likely secondary to neuroplasticity changes.

The brain area to be targeted is another crucial aspect in setting the rTMS for W’sC (Antelmi et al., 2017; Erro et al., 2017). Previous studies converged on the PMC as a potentially useful candidate for rTMS, but there is not yet clear evidence on the neurophysiological mechanisms (Erro et al., 2017; Quartarone et al., 2017). In fact, there was too much experimental group heterogeneity (e.g., both W’sC and musicians’ dystonia) in the previously performed studies to draw any clear conclusion (Kimberley et al., 2013, 2015). Even though the study is a single-case report, our data suggest that low-frequency rTMS over the PMC is able to revert both the abnormal sensorimotor plasticity and the lack of topographical specificity of muscle activation that characterize W’sC pathophysiology, thus improving motor symptoms. The large effects of rTMS on sensorimotor plasticity and topographical specificity of muscle activation, we found may have occurred through a low-frequency induced reduction of an abnormally high excitatory output going from the PMC to the sensorimotor regions (through cortico-basal ganglia-cortical loops), as indicated by some studies employing TMS and EEG (Teulings, 1996; Siebner et al., 1999; Paus et al., 2001; Strafella et al., 2001; Houdayer et al., 2008; Borich et al., 2009; Kantak et al., 2012; Bharath et al., 2015, 2017; Haith et al., 2016; Longcamp et al., 2016; Antelmi et al., 2017). This is particularly relevant during handwriting in patients with focal dystonia. In fact, PMC sustains a greater activation of left intraparietal sulcus, right cerebellum, left anterior putamen during initiation of handwriting, and of left ventral PMC and inferior and superior parietal cortices during handwriting as compared to non-handwriting motor tasks (Odergren et al., 1998; Lerner et al., 2004), which has been reverted by using rTMS on PMC (Wolters et al., 2003). It could be indeed interesting to compare PMC and primary sensorimotor areas in terms of method effectiveness, even though the latter influence a smaller number of brain regions within the cortical and subcortical motor system, also during handwriting (Chouinard et al., 2003).

Our study has three main limitations: first, we cannot confidently exclude a placebo effect, unless replicating the results in a group of patients with a sham-controlled study design. Notwithstanding, previous placebo- or case-controlled rTMS studies in patients with W’sC revealed no clinical changes in the placebo/sham-TMS control group, thus suggesting the factual efficacy of PMC rTMS (Siebner et al., 1999; Murase et al., 2005; Borich et al., 2009; Kimberley et al., 2013, 2015; Nordmann et al., 2015; Erro et al., 2017). However, further studies remain necessary to definitely prove PMC low-frequency rTMS efficacy as the results were still not deterministic. The lack of an MRI-based neuronavigation system in the experiment is the second major limitation of the study. Last, one may argue that the neurophysiological effects, we found may have been biased by an interference between rPAS and 1 Hz rTMS by means of, e.g., metaplasticity phenomena, given that both of them are a form of plasticity-inducing protocol. However, rPAS effects were measured only before and immediately after the entire rTMS protocol, which lasted many weeks. So, the fact that an interaction between the two protocols may have sustained the treatment effect seems unlikely. This was also the reason why we did not precautionally perform any rPAS measurement during 1 Hz rTMS treatment.

## Conclusion

To date, a clear evidence regarding low-frequency rTMS as therapeutic tools for W’sC is still lacking, and therefore rTMS is actually considered a complementary rather than an alternative treatment. Even though the low frequencies reduce cortical excitability, rTMS effects vary depending on the characteristics of individual excitability and the stimulation setup. Consequentially, further studies are necessary to confirm our promising data and to better characterize the neurophysiological basis underlying rTMS-induced clinical improvement. Nonetheless, our case suggests a potential efficacy of long-duration 1 Hz rTMS to PMC of the hemisphere contralateral to the affected hand to improve W’sC symptoms. It remains to be confirmed whether low-frequency PMC rTMS can reliably revert the maladaptive sensory-motor plasticity mechanisms and improve the topographical specificity of muscle activations, which are among the basic neurophysiological abnormalities characterizing W’sC.

## Data Availability

All datasets generated for this study are included in the manuscript.

## Ethics Statement

The studies involving human participants were reviewed and approved by IRCCS Centro Neurolesi Bonino Pulejo. The patients/participants provided their written informed consent to participate in this study.

## Author Contributions

AN: manuscript draft writing. LB: manuscript draft writing and experiment execution. SP: experiment execution. PB and RC: critique review.

## Conflict of Interest Statement

The authors declare that the research was conducted in the absence of any commercial or financial relationships that could be construed as a potential conflict of interest.
